# Case Report: 3D imaging-assisted minimally-invasive hybrid closure surgery of a complex coronary artery fistulas

**DOI:** 10.3389/fcvm.2024.1439263

**Published:** 2024-11-22

**Authors:** Yu Chen, Jin Lu, Xingchen Lian, Peipei Chang, Ping Wen, Lin Ma, Yuhang Liu

**Affiliations:** ^1^ Department of Cardiothoracic Surgery, Dalian Municipal Women and Children’s Medical Center(Group), Dalian, China; ^2^ Graduate School, Dalian Medical University, Dalian, China; ^3^ Beijing Children’s Hospital Heilongjiang Hospital, Heart Center, Haerbin, China

**Keywords:** coronary artery fistula, congenital heart disease, three-dimensional imaging, minimally invasive hybrid closure surgery, case report

## Abstract

Coronary artery fistulas (CAFs) are rare congenital heart defects that are typically managed through interventional closure, traditional surgery, or minimally invasive hybrid closure surgery. However, treating CAFs with complex anatomy, such as tortuous vessels, presents a significant challenge, particularly in young children. We report the case of a 3.8-year-old child (15 kg/100 cm) with a complex CAF, treated using a minimally invasive hybrid closure surgery approach with a 4  ×   4 mm Amplatzer Duct Occluder II (ADO II) (Abbott, USA). Three-dimensional (3D) imaging was utilized to visualize the CAF's anatomy, guide the surgical planning, and accurately determine the puncture site on the right ventricular free wall, as well as the optimal sheath direction and insertion depth. The procedure was carried out efficiently and safely, guided by preoperative 3D imaging and intraoperative transesophageal echocardiography. Follow-up at one year demonstrated excellent outcomes with no complications.

## 1 Introduction

Coronary artery fistulas (CAFs), which account for approximately 0.2%–0.4% of all congenital heart diseases (1, 2), are very rare coronary artery malformations, occurring with an incidence of 0.1%–0.22%. They generally do not present with clinical signs or symptoms and are usually discovered incidentally. However, the likelihood of their spontaneous closure is very small, and large and/or symptomatic CAFs have an extremely high chance of developing late symptoms and complications. Therefore, early treatment is necessary once a CAF is diagnosed (3, 4). Treatment methods for CAFs include interventional closure, traditional surgery, and minimally invasive hybrid closure surgery. Traditional surgery is suitable for almost all CAFs, whereas interventional closure is more suited for patients with a more accessible fistula or straight fistula course. However, traditional surgery and interventional closure are more challenging for CAFs that have complex anatomical structures and deep anatomical locations and are difficult to expose. Herein, we report a case of a patient with a CAF who was treated with minimally invasive hybrid closure surgery, which was successfully performed under the guidance of preoperative three-dimensional (3D) imaging and intraoperative transesophageal echocardiography (TEE).

## 2 Case report

**Figure 1:**
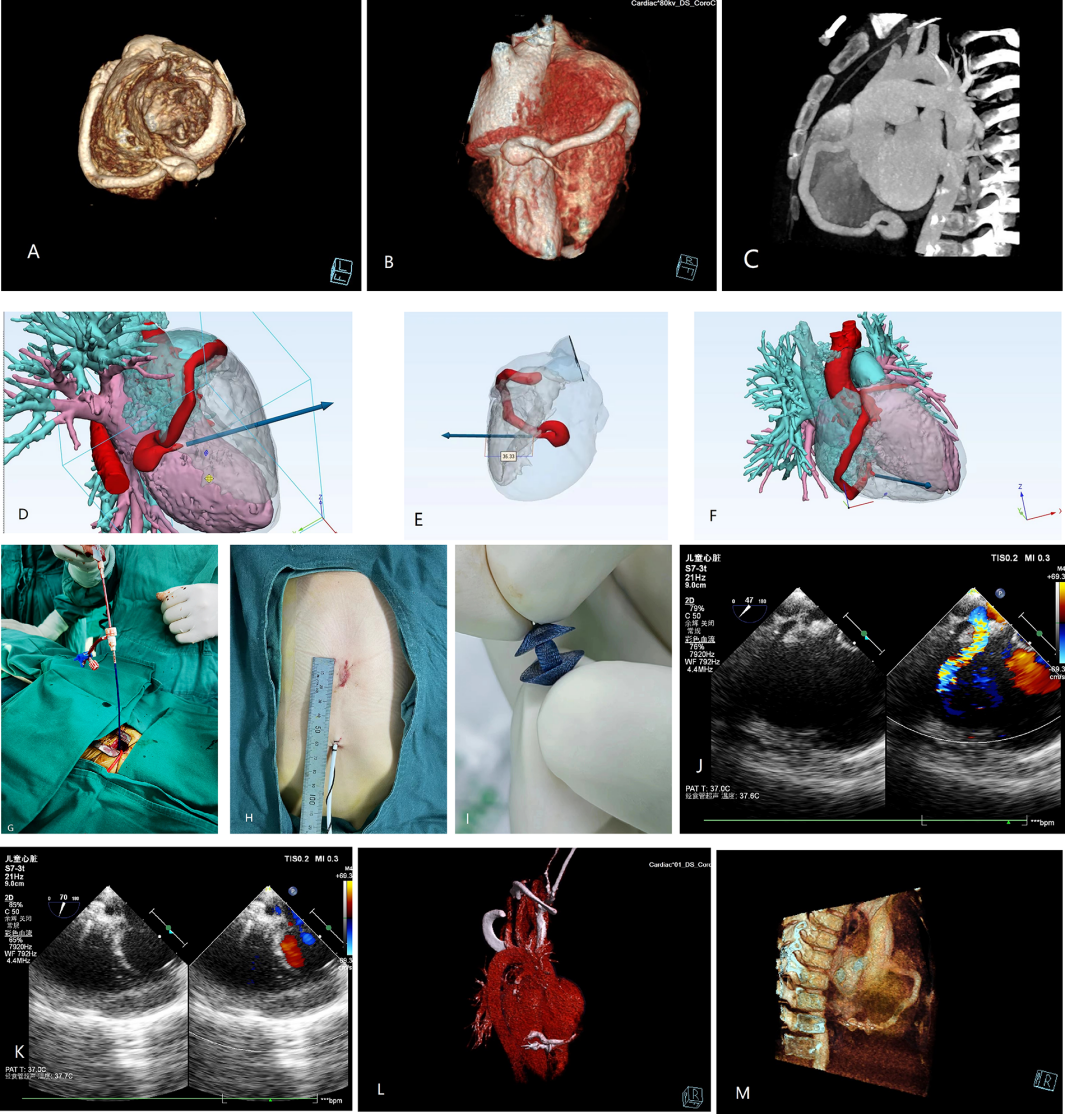
Preoperative 3D **(A,B)** and 2D **(C)** computed tomography (CT) images. **(D–F)** 3D images simulating the fistula drainage position and allowing reverse extrapolation of the puncture site and depth on the right ventricular free wall. **(G)** Surgical procedure. **(H)** Access incision. **(I)** Occluder. **(J,K)** Intraoperative ultrasound image for identifying the direction of blood flow and determining the direction of sheath guidance. Postoperative 3D **(L)** and 2D **(M)** CT images of the successfully occluded coronary artery fistula.

A 2.8-year-old boy was referred to our institution for a grade III/IV systolic heart murmur detected during a physical examination for pneumonia. Echocardiography revealed slight enlargement of the left heart and dilation of the right coronary artery (RCA), with an internal diameter of approximately 5 mm. A 4 mm CAF was observed in the apical part of the right ventricle (RV), with a left-to-right shunt, and a maximal continuous flow velocity of 4.4 m/s. Cardiac computed tomography angiography (CTA) (Figure 1A–C) confirmed that the RCA was dilated giving birth to a CAF extending from the atrioventricular groove to the base of the heart, making a 360° rotation before draining into the RV, with a posterior descending branch evident at the lateral wall of the fistula. Preoperative simulation using computer-based 3D imaging (Figure 1D–F) was performed to determine the puncture site on the right ventricular free wall, as well as the sheath direction and insertion depth. The case was discussed during a multidisciplinary meeting and a decision for elective minimally invasive hybrid surgery was taken at the age of 3.8 years old (15 kg/100 cm).

### 2.1 Surgical procedure

After obtaining the parents' consent, the procedure was undertaken in the operating theater under transesophageal echocardiography (TEE) guidance. After systemic heparinization and antibiotic prophylaxis, a 2 cm incision was made at the inferior medial end of the sternum, with dissection of the inferior sternum layer by layer. The pericardium was incised and suspended to expose the anterior wall of the RV. The puncture site was identified using a combination of preoperative 3D imaging and intraoperative TEE (Figure 1D–F, Figure 1J–K). A purse-string suture was placed at the puncture site, followed by puncture and insertion of the guidewire (4-F) and sheath (1.17 mm). The TEE operator guided the sheath into the fistula, and a 4  ×  4 mm Amplatzer Duct Occluder II (ADO II) (Abbott, USA) was inserted along the sheath until it was implanted. The occluder was released after a multi-view ultrasound confirmed no residual shunt or abnormalities (Figure 1G–K). The procedure lasted 30 min. The patient was extubated on the first postoperative day and discharged on the fourth postoperative day under oral heparin. Repeat examinations showed normal results (Figure 1L, M). Follow-up at 1 month, 2 months, 6 months, and 1 year revealed the occluder was well-positioned with no significant abnormalities observed.

## 3 Discussion

CAFs are a very rare congenital heart disease. Small CAFs may close spontaneously over time, whereas medium and large fistulas may expand and lead to complications such as coronary artery dilatation, aneurysm, atherosclerosis, myocardial infarction, and cardiac arrhythmia (2, 5, 6). Therefore, early treatment is recommended once diagnosed (7).

Traditional surgery and interventional closure are the main methods used to treat CAFs (8). However, traditional surgery involves performing a sternotomy, with some patients requiring extracorporeal circulation, which is highly traumatic and prone to risk of complications. Although interventional closure does not require a sternotomy or extracorporeal circulation (2), it has certain anatomical structure requirements (4, 9), and shortcomings such as radiation damage, vascular damage, and contrast allergy can occur intraoperatively. Furthermore, it carries a chance of postoperative complications, such as device displacement, myocardial infarction, and thrombosis (4). With recent advances in medical technology, minimally invasive hybrid closure has been increasingly employed in cardiac surgery, with studies showing it to be a safe and effective treatment approach for CAFs (10). Not only does the procedure avoid the need for extracorporeal circulation and x-ray irradiation, but it also has a wider scope of application and greatly reduces the occurrence of intraoperative and postoperative complications (3, 10, 11). However, minimally invasive hybrid closure procedure demands a high level of proficiency frolm the operator along with meticulous of the appropriate occluder. Physicians must carefully choose the size and model of the device based on the patient's unique condition, ensuring precise delivery to the defect site during the procedure. The timing and location of device release are critical for a successful outcome. While the procedure offers benefits such as reduced trauma and faster recovery, it is not without risks. potential complications may include device detachment, migration, thrombosis formation, and cardiac perforation. In our patient, the right coronary artery was dilated, extending from the atrioventricular groove to the base of the heart and turning a 360° rotation before draining into the right ventricle, with the posterior descending branch positioned at the lateral wall of the fistula. Because both the traditional surgical treatment and interventional closure options would have led to difficulties in this young patient, we decided that minimally invasive hybrid closure would be more suitable for him and performed the preoperative evaluation of cardiac anatomy using 3D imaging.

Three-dimensional imaging refers to the computer-aided design of 3D digital models that can simulate the visual experience of the human eye for real-world objects, allowing images or videos to be captured on visual media. Since we can only parse images on a two-dimensional level and lack 3D imagination, our ability to grasp anatomical details to achieve fine operation is limited. Therefore, we used 3D imaging to guide the surgery, which not only enabled preoperative simulation and surgical training but also helped the patient's family to better understand the treatment options and surgical methods during the communication process and gain an understanding of our clinical work. Additionally, 3D imaging allows us to tailor personalized anatomical structures for the patient, observe anatomical details more intuitively and accurately, and attain a good basis for surgical planning and surgical simulation (12, 13). This technology has also been applied more extensively for the guidance of cardiac surgery. Nazario (2022) reported the use of 3D printing in the surgical treatment of left ventricular aneurysms in 2022 (14). In our case, we combined this technique with preoperative cardiac CTA to construct the 3D anatomical structure of the heart as well as clarify the origin, course, and location of the CAF, construct a coordinate system on 3D imaging, reversely extrapolate the location of the puncture site, predict the sheath guidance direction and insertion depth, and formulate a surgical plan preoperatively. These actions enabled the safe and efficient implementation of the procedure.

Three-dimensional imaging-assisted minimally invasive hybrid closure can successfully be used to treat anatomically complex CAFs. To the best of our knowledge, this is the first report on the successful implementation of 3D imaging-assisted hybrid closure for a CAF in a pediatric patient. Using preoperative 3D imaging, we were able to intuitively visualize the location of fistula drainage, infer the direction of blood flow, reverse extrapolate the puncture site on the right ventricular free wall, and predict the direction of sheath guidance, thereby avoiding blind penetration of the puncture site without first clarifying the direction of puncture during the surgical process. When combined with intraoperative TEE, we were able to rapidly identify the fistula and predict the depth of sheath insertion, thereby preventing complications arising from damage to the posterior ventricular wall and coronary artery due to excessively deep sheath insertion.

## 4 Conclusion

For CAFs with complex anatomical structures, such as tortuous courses, preoperative 3D imaging to visualize cardiac anatomy can significantly enhance the success of minimally invasive hybrid closure procedures and reduce the risk of complications in small children. This technique holds great potential for improving outcomes and should be further promoted in clinical practice.

## Data Availability

The original contributions presented in the study are included in the article/Supplementary Material, further inquiries can be directed to the corresponding author/s.
