# Supplementary figures and images for: Case Report: 3D imaging-assisted minimally-invasive hybrid closure surgery of a complex coronary artery fistulas
Source: Front Cardiovasc Med. 2024 Nov 22;11:1439263. doi: 10.3389/fcvm.2024.1439263 (PMC11621213; doi:10.3389/fcvm.2024.1439263)

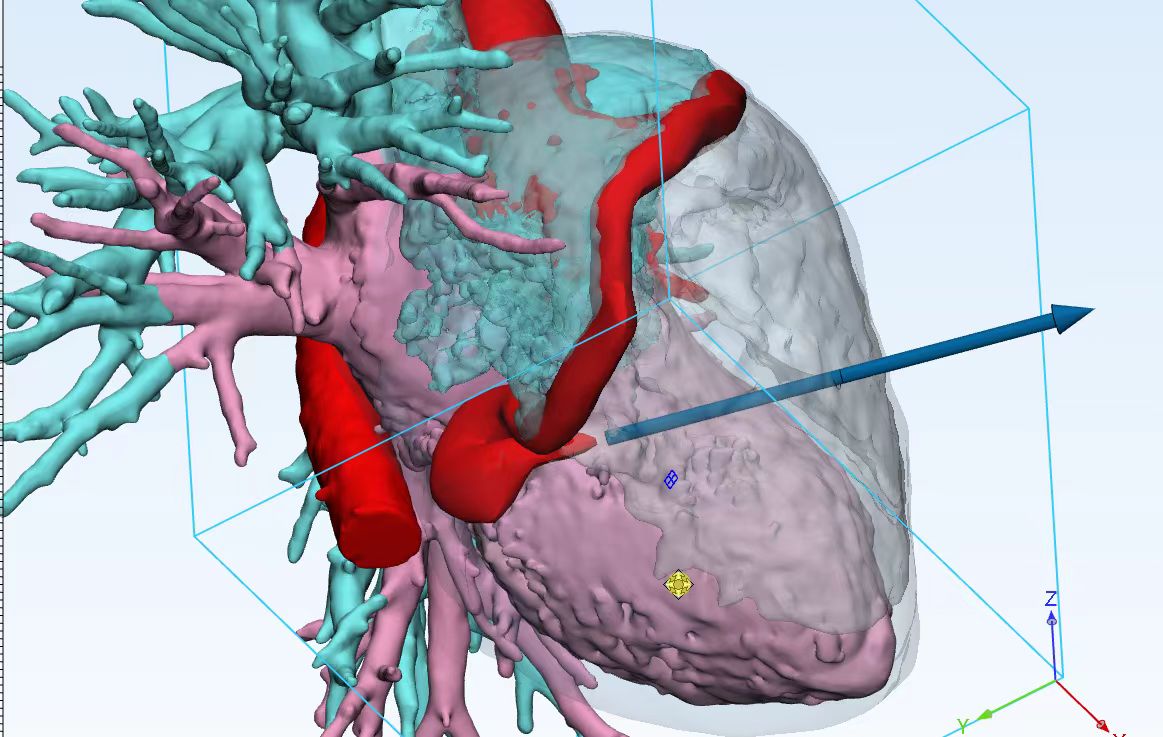

Supplement: Supplementary file 1 [file Image1.jpeg]

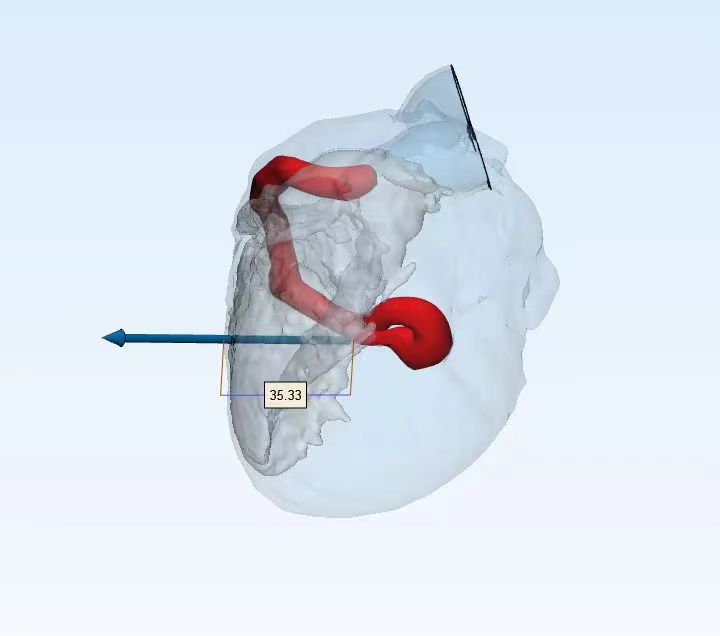

Supplement: Supplementary file 2 [file Image2.jpeg]

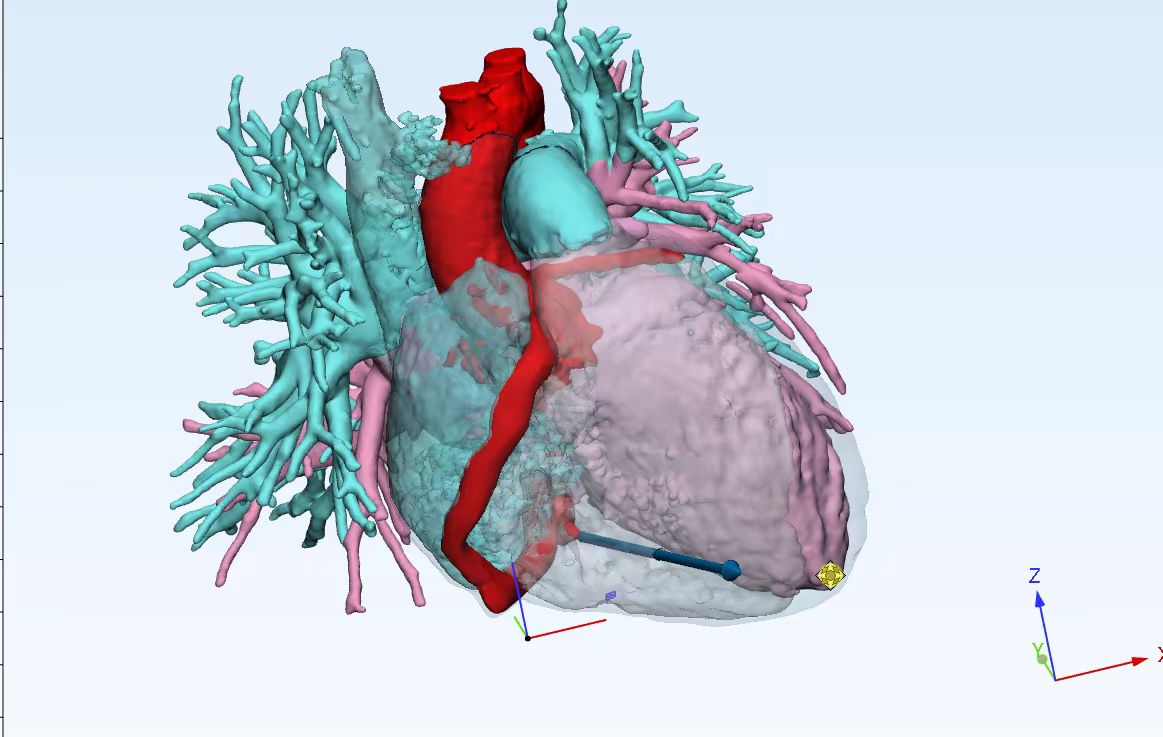

Supplement: Supplementary file 3 [file Image3.jpeg]

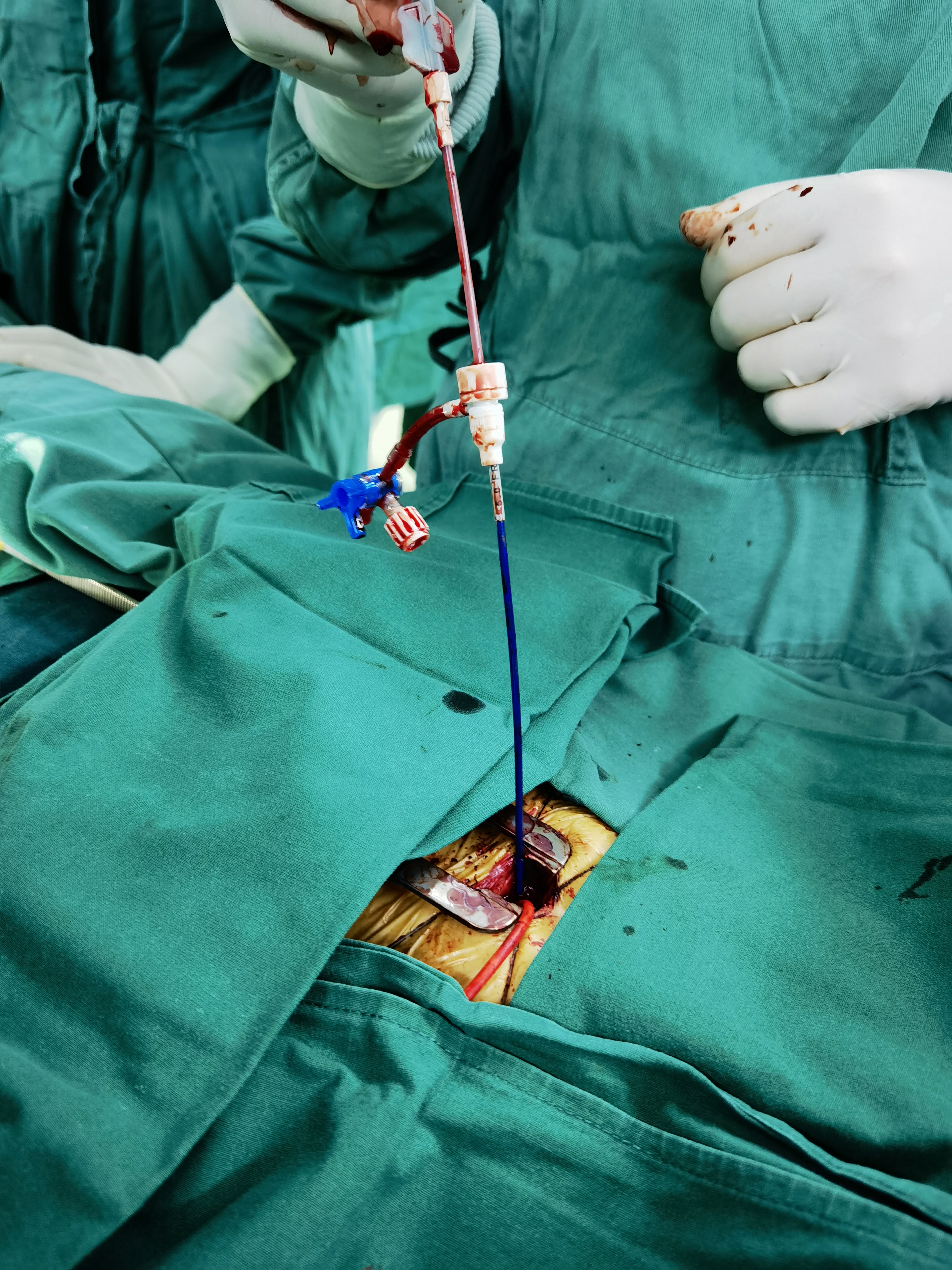

Supplement: Supplementary file 4 [file Image4.jpeg]

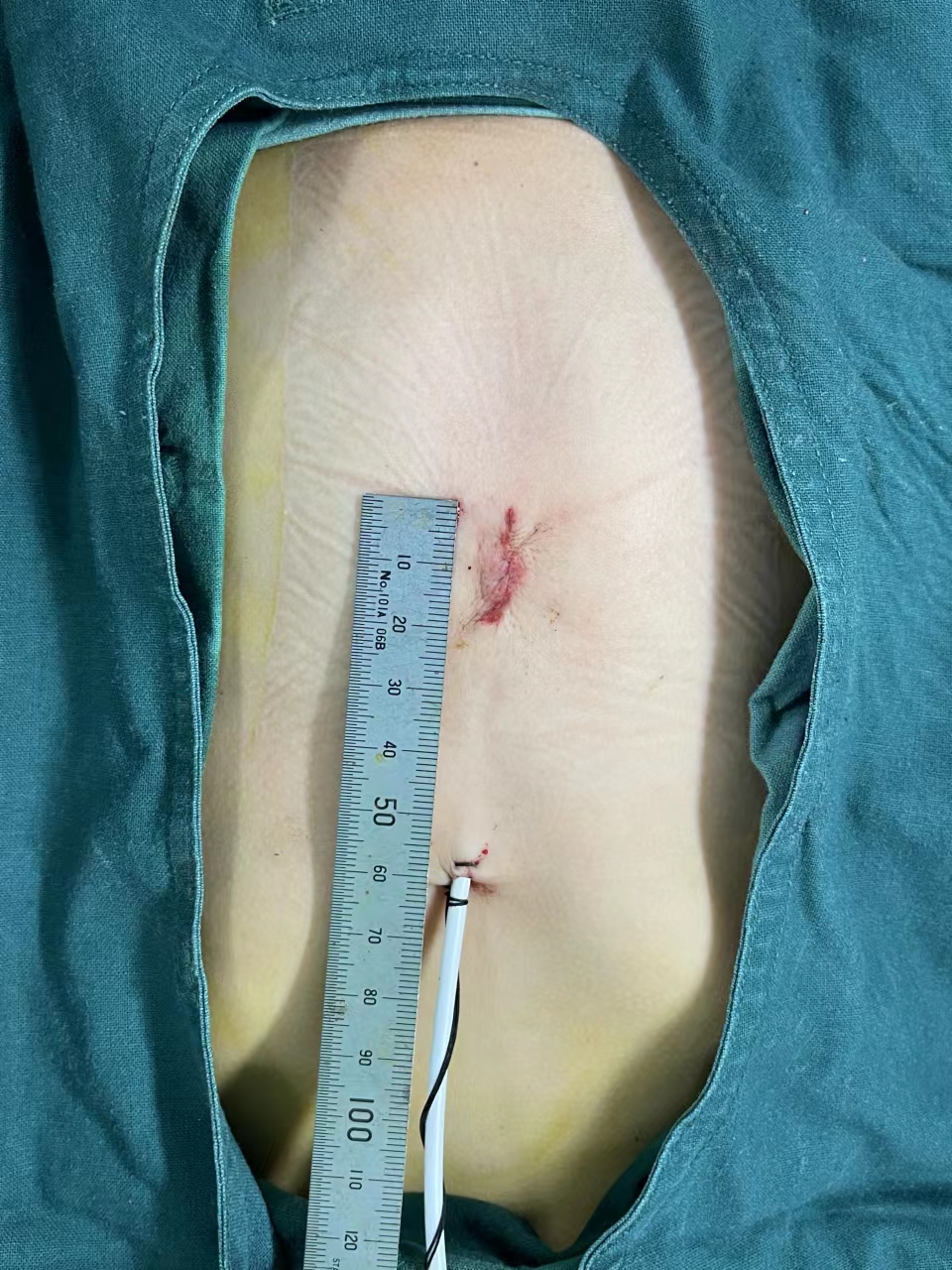

Supplement: Supplementary file 5 [file Image5.jpeg]

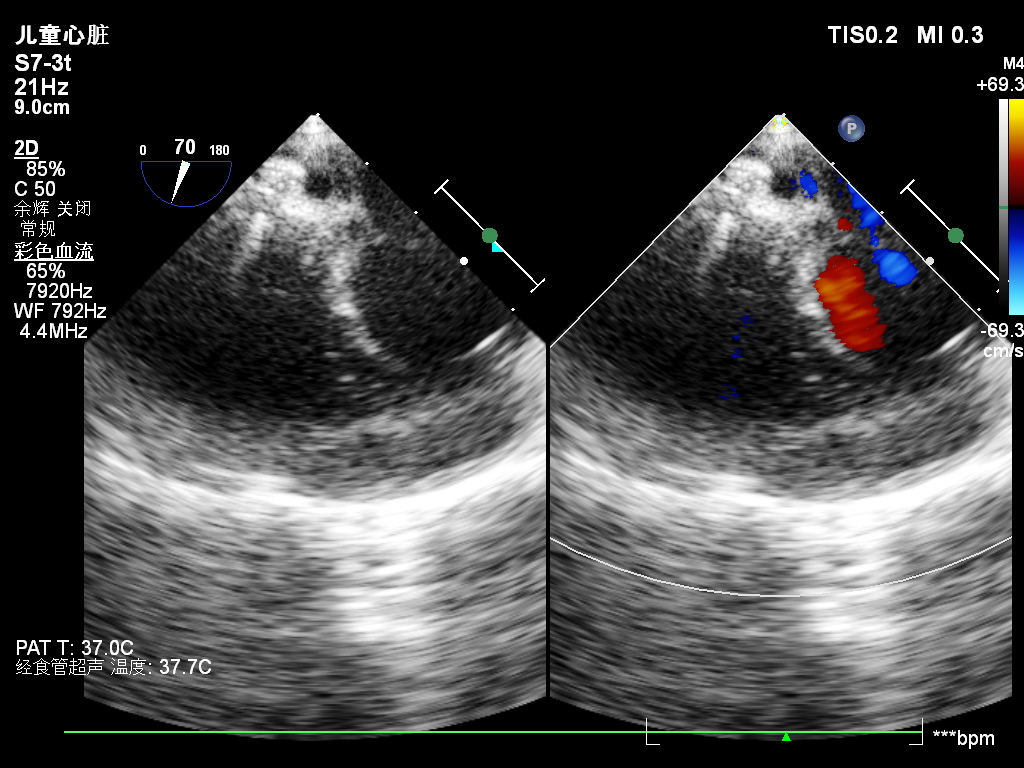

Supplement: Supplementary file 7 [file Image7.jpeg]

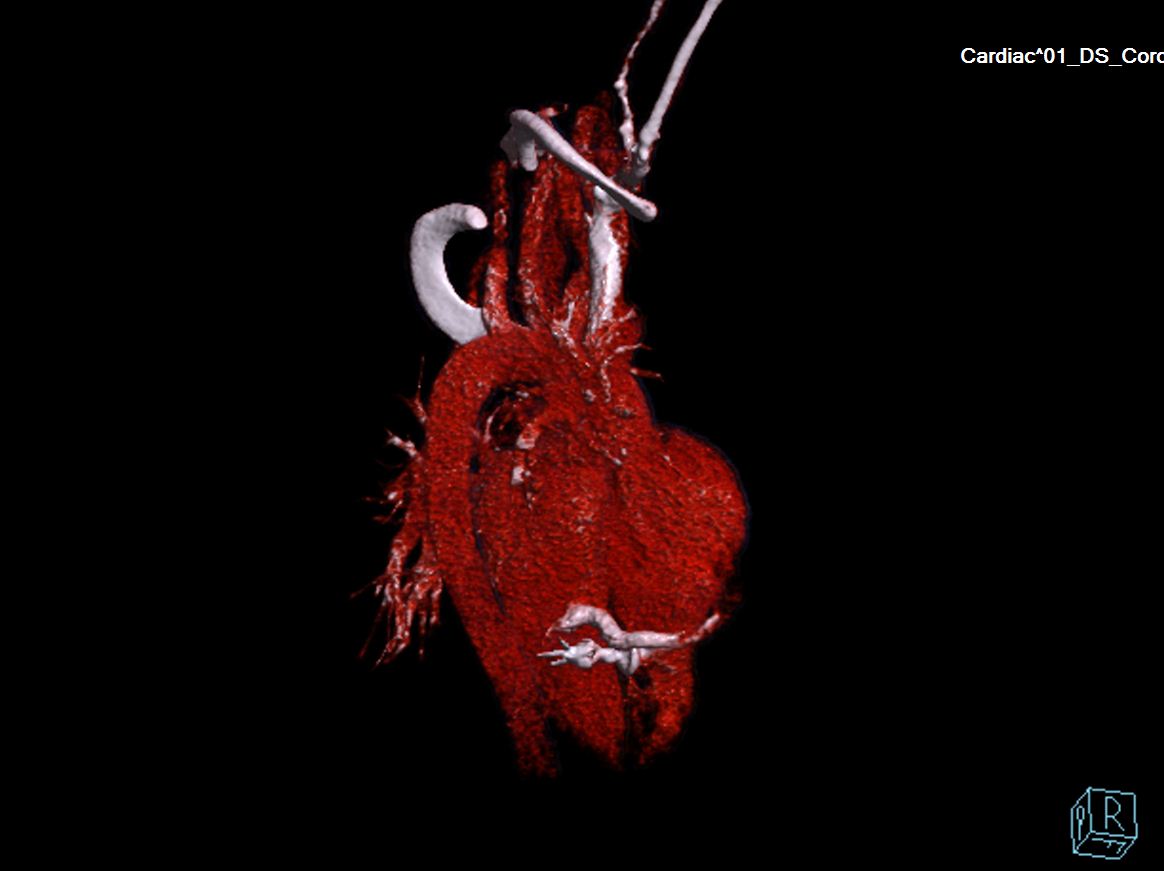

Supplement: Supplementary file 8 [file Image8.jpeg]

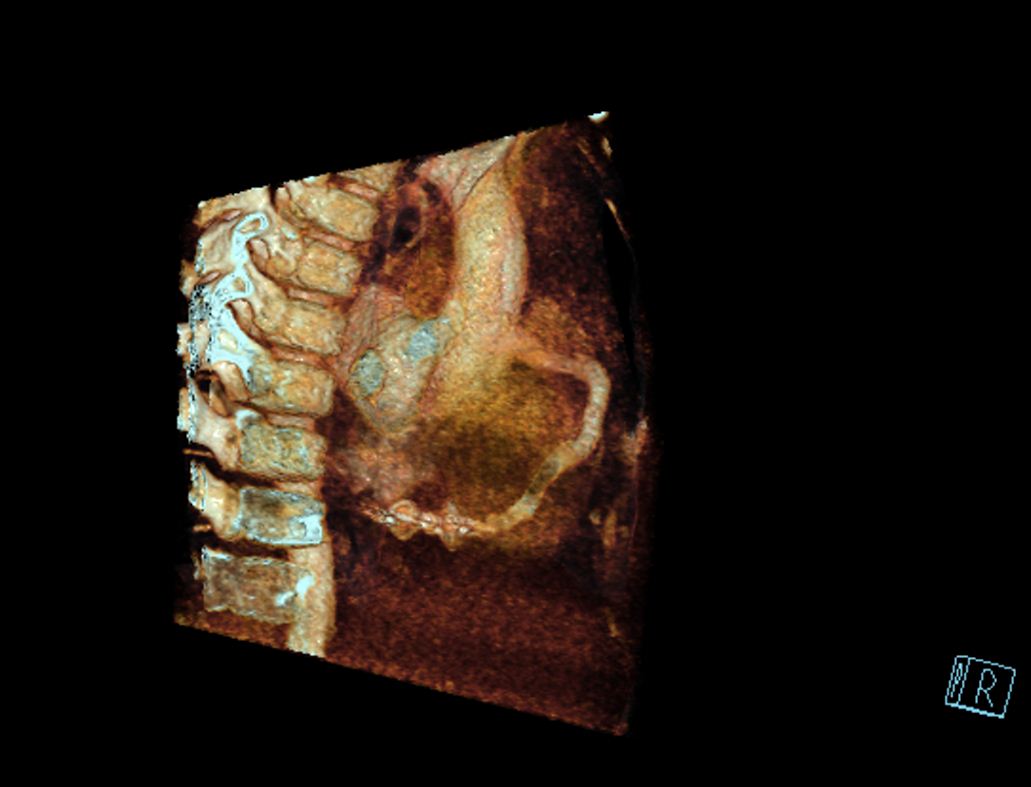

Supplement: Supplementary file 9 [file Image9.jpeg]
